# Supplementary material for: Factors affecting anxiety among administrative officers working within the urgent protective action planning zone of a nuclear power station
Source: PLoS One. 2020 Aug 5;15(8):e0236997. doi: 10.1371/journal.pone.0236997 (PMC7406078; doi:10.1371/journal.pone.0236997)
Supplement: S1 File — (DOCX) [file pone.0236997.s001.docx]

原子力発電所UPZ区域内の行政職員の皆様へ

質問への回答にご協力をお願いします。あてはまる番号に○をつけ、(　)内に回答を記入して下さい。

質問1　性別を教えて下さい。　　　① 男性　　　　　② 女性

質問2　年代を教えて下さい。

➀ 20代　② 30代　③ 40代　④ 50代　⑤ 60代　⑥ 70歳以上

質問3　何人暮らしですか？　　　① 独居　　　　　② それ以外

質問4　15歳以下のお子様がいますか？　　　① いる　　　　　② いない

質問5　現在の居住形態はどうですか？　　　➀　持ち家　　　　　② 借家

質問6　どちらにお住まいですか?川内原子力発電所から概ね半径、

➀ 5km内　② 10km内　③　20km内　④　30km内　⑤　30km外　⑥　わからない

質問7　そちらでの居住年数を教えて下さい。(　　　　　)年

質問8　そちらでの勤続年数を教えて下さい。(　　　　　)年

質問9　職位を教えて下さい。　　　①　一般職員　　　　　② 係長級以上

問題10　あなたの職種は原子力防災関係ですか？　　　① はい　　　　　② いいえ

質問11　現在の部署での勤続年数を教えて下さい。(　　　　　)年

質問12　あなたの職務内容では、住民から、原子力関連の相談を受ける機会がありますか？

① ある　　　　　② ない

質問13　地域防災計画(原子力災害関連)についてご存知ですか？

①　知っている　　②　知らない

質問14　地域防災計画(原子力災害関連)の内容をご存じですか？

➀　よく知っている　　② 知っている　　③ あまり知らない　　④ 全く知らない

質問15　原子力防災訓練に参加したことがありますか？

① はい　　　　　② いいえ

質問16　現在、原子力発電所UPZ区域内で勤務している事で不安を感じますか？

①　大いに不安を感じる　　②　やや不安を感じる　　③　あまり不安を感じない　④　全く不安を感じない

質問17　医療機関で、胸部レントゲン、CT、心臓カテーテル、マンモグラフィーなどの放射線を利用した検査を受けることに抵抗がありますか？

① ある　　　　　② ない

質問18　放射線防護の3原則を知っていますか？

① 知っている　　　　　② 知らない

質問19　放射線の種類に「自然放射線」と「人工放射線」があることを知っていますか？

➀　知っている　　　　　② 知らない

質問20　シーベルトとベクレルの単位の違いを知っていますか？

① 知っている　　　　　② 知らない

質問21　「放射性物質」に半減期があることを知っていますか？

① 知っている　　　　　② 知らない

質問22　放射線には外部被ばくと内部被ばくがあることを知っていますか？

① 知っている　　　　　② 知らない

質問23　一般市民には、年間放射線量限度が設けられていることを知っていますか？

① 知っている　　　　　② 知らない

質問24　「安定ヨウ素剤」を知っています？

① 知っている　　　　　② 知らない

以上でアンケートは終わりです。ご協力ありがとうございました。
