# Supplementary material for: Factors affecting anxiety among administrative officers working within the urgent protective action planning zone of a nuclear power station
Source: PLoS One. 2020 Aug 5;15(8):e0236997. doi: 10.1371/journal.pone.0236997 (PMC7406078; doi:10.1371/journal.pone.0236997)
Supplement: S2 File — (DOCX) [file pone.0236997.s002.docx]

**Questionnaire**

Please answer the following questions and circle that apply.

1. Sex: 1) Male 2) Female

2. Age:　 1) 20's　　2) 30's　　3) 40's　　4) 50's　　5) 60's　　6) 70's

3. How many people are you living in? 1) Single household 2) 2 people or more

4. Are you living with children younger than 15 years? 1) Yes 　　 2) No

5. Are you living in own house? 1) Yes 　　 2) No

6. How many radius do you live in from the nuclear power plant?

1) 5km　　2) 10km　　3) 20km　　4) 30km 　　5) 30km over　　6) I do not know

7. How many years are you living in this area? (　　　 )years

8. How long have you been working in this area? ( )years

9. What is your current position? 1）General staff　　2）Principal staff

10. Are you affiliated with nuclear disaster prevention? 1) Yes 　　 2) No

11. How many years are you working in your current department? (　　　 ) years

12. Have you ever received questions about radiation from residents? 1) Yes 2) No

13. Do you know about regional plan for nuclear disasters prevention? 1) Yes 2) No

14. Do you know your own role in the regional plan for nuclear disasters prevention?

1) Yes 2) No

15. Have you ever participated in nuclear disaster drills? 1) Yes 2) No

16. Have you ever experienced anxiety about daily life while working within the Urgent Protective Action Planning Zone?

1)　Yes　　 　2)　Probably 3)　Probably no　　4)　No

17. Are you reluctant to undergo a radiological examination at a hospital? 1) Yes 2) No

18. Do you know the three principles of radiation protection? 1) Yes 2) No

19. Do you know types of radiation? 1) Yes 2) No

20. Do you know unites of radiation? 1) Yes 2) No

21. Do you know half-dacay of radionuclides? 1) Yes 2) No

22. Do you know internal and external radiation exposure? 1) Yes 2) No

23. Do you know annual radiation dose limit of the public? 1) Yes 2) No

24. Do you know stable potassium iodide? 1) Yes 2) No

Thank you for your cooperation.
