# Supplementary material for: Factors affecting anxiety among administrative officers working within the urgent protective action planning zone of a nuclear power station
Source: PLoS One. 2020 Aug 5;15(8):e0236997. doi: 10.1371/journal.pone.0236997 (PMC7406078; doi:10.1371/journal.pone.0236997)
Supplement: Attachment — Submitted filename: 200626ResponseToReviewers.docx [file pone.0236997.s004.docx]

**Author Response to Editorial Comments:**

*I appreciate the amount of work the authors have put into this study, which is an important one given the lacunae in this area. There are a few minor issues to be addressed in this manuscript moving forward: in particular I hope the authors put extra focus on responding to Reviewer 1's comments on the somewhat problematic usage of "maternal characteristics of women" that appears throughout the manuscript. While I think many individuals may appreciate the innocuous sentiment behind the statement, the authors need to rephrase this or qualify the statement: are they referring to the societal duties imposed upon women, etc.?*

We greatly appreciate your comments on our manuscript and provide point-by-point responses to the issues raised by the reviewers below. We have revised some sections of the text and added more information. In particular, according to your suggestion, we revised the text that reviewer 1 commented on as follows: “We indicated that females expressed greater concern about the health effects of radiation exposure than males, which is consistent with previous studies [7, 8]. This might be due to women’s motherly instincts, such as feeling anxiety about childbearing and raising children [7, 8, 13, 14]” (Page 14, Lines 175 – 178).

**Author Response to Journal Requirements:**

We greatly appreciate your comments on our manuscript. We have revised the manuscript according to your suggestions.

*1. Please ensure that your manuscript meets PLOS ONE's style requirements, including those for file naming. The PLOS ONE style templates can be found at here.*

We adjusted our formatting to ensure that our manuscript meets PLOS ONE’s style requirements including adding the captions for the Supporting information files at the end of the manuscript (Title page, Reference and Page 19, Lines 314 – 320).

*2. Please include additional information regarding the survey or questionnaire used in the study and ensure that you have provided sufficient details that others could replicate the analyses. For instance, if you developed a questionnaire as part of this study and it is not under a copyright more restrictive than CC-BY, please include a copy, in both the original language and English, as Supporting Information. In addition, please provide any details of the pre-testing of this questionnaire that took place.*

We added a copy of the questionnaire used in this study, in both the original language (Japanese) and English, as Supporting information Files S1 and S2. We added the following text in the Materials and methods section: “The questionnaire used in this study was developed based on questionnaires used in previous studies conducted in Fukushima Prefecture on intention to leave work [7, 8] and on the mental health and risk perception survey within the framework of the Fukushima Health Management Survey, which was organized by Fukushima Prefecture [4]. The questionnaire was verified with the confirmation of the local government office and Kagoshima University Graduate School of Health Sciences (No. 324). The original questionnaire form is provided in Supporting information File S1 and File S2.” (Page 5, Lines 98 – 104.)

*3. Please include captions for your Supporting Information files at the end of your manuscript, and update any in-text citations to match accordingly.*

We added the captions for the Supporting information files at the end of the manuscript and updated references to these files in the text accordingly. (Page 5, Lines 103 – 104; Page 6, Lines 122 – 123; Page 19, Lines 314 – 320.)

**Author Response to Reviewer.**

**Reviewer #1:**

*The article is an important contribution to the field. A few suggested edits are below:*

We greatly appreciate your comments on our manuscript. We have revised the manuscript according to your suggestions.

*- The background primarily focuses on radiation exposure, but the article is focused on risk perception. Revising this section to highlight some data on risk perception would be more congruent with the paper.*

According to your suggestions, we revised the Introduction section. The description of radiation exposure is summarized as follows: “These measurements of external and internal radiation exposure of residents surrounding the FDNPS have been reported by several research institutions; they all suggest that external and internal radiation doses caused by the accident were relatively low and far from any direct health consequences in the general population [3].” (Page 3, Lines 38 – 41.)

Text describing individual risk perception after the Fukushima accident and the relevant citations were added as follows: “Various information was transmitted to the public regarding the radiation-related health risk following the accident and many people in Fukushima felt anxiety about the health effects of radiation exposure, which resulted in social panic. The Fukushima Health Management Survey revealed that among evacuees of the Fukushima disaster, psychological distress was more frequent among people who perceived health effects of radiation exposure to be very likely, it appears that psychological status was related to the perception of radiation risks [4]. This result suggests that incorrect understanding of health effects of radiation may be related to psychological distress [5]. Furthermore, this anxiety was felt even in communities well outside of Fukushima [6].” (Page 3, Lines 42 – 50.)

*- The authors speculation on causes of significant findings requires more information. Particularly line 164 discussing the potential causal factor being "the maternal characteristics of women". This needs more explanation. The citations are also focused on burnout of nurses in the context of occupational stress and burnout.*

According to your suggestion, we revised the text as follows: “We indicated that females expressed greater concern about the health effects of radiation exposure than males, which is consistent with previous studies [7, 8]. This might be due to women’s motherly instincts, such as anxiety about childbearing and raising children [7, 8, 13, 14].” (Page 14, Lines 175 – 178).

*- The conclusions to build a comprehensive education program should take into account the degradation in trust in government authorities after the 3/11 disasters. Some acknowledgement of the research in this regard could also be cited in the conclusions and/or in the study limitations.*

According to your suggestion, we added the following to the conclusion section: “Also, it is necessary for administrative officers to build their credibility with the public through daily communication, and to disseminate accurate information about radiation [21]. From this point of view, it is important for administrative officers to develop a comprehensive educational program to build trust with the public and reduce public concerns about radiation exposure and its health effects with the cooperation of radiation medical science experts.” (Page 15, Lines 201 – 207). In addition, we have also added the following sentence as a study limitation: “…we could not obtain sufficient information on potential confounding factors, such as detailed lifestyle habits, educational environment, the degradation in trust in the central government after the Fukushima disasters and the responsibilities of study participants related to nuclear disasters.” (Page 15, Lines 212 – 216).

**Reviewer #2:**

*The paper had been able to comply with the standards of the Journal. However, some few points need to be define further.*

We greatly appreciate your comments on our manuscript. We have revised the manuscript according to your suggestions.

*1. How did they validate the questionnaires used in this study?*

According to your suggestion, we revised the Materials and Methods section as follows: “The questionnaire used in this study was developed based on questionnaires used in previous studies conducted in Fukushima Prefecture on intention to leave work [7, 8] and on the mental health and risk perception survey within the framework of the Fukushima Health Management Survey, which was organized by Fukushima Prefecture [4]. The questionnaire was verified with the confirmation of the local government office and Kagoshima University Graduate School of Health Sciences (No. 324). The original questionnaire form is provided in Supporting information File S1 and File S2.” (Page 5, Lines 98 – 104.)

*2. For the population sampling, how did you choose your study subjects?*

According to your suggestion, we revised the Materials and Methods section as follows: “We distributed questionnaires to all 1,558 general administrative officers who worked at local public offices within the UPZ of Sendai Nuclear Power Station (SNPS) in Kagoshima Prefecture (Fig 1), which was the first nuclear power station restarted after the FDNPS accident in Japan. The 1,181 (75.8%) officers who provided complete responses to the questionnaires were included in the analysis.” (Page 4 and 5, Lines 84 – 89.)

*3. Some few grammatical errors had been observed.*

According to your suggestion, a native English speaker checked the grammar in the revised manuscript.

**Reviewer #3:**

*There are not many studies on radiation anxiety related with nuclear plant disaster in Fukushima. This is a good research with technical soundness, appropriate statistical analysis, sharing of relevant data in the paper and written in standard English.*

We greatly appreciate your comments on our manuscript.
