# Supplementary material for: Factors affecting anxiety among administrative officers working within the urgent protective action planning zone of a nuclear power station
Source: PLoS One. 2020 Aug 5;15(8):e0236997. doi: 10.1371/journal.pone.0236997 (PMC7406078; doi:10.1371/journal.pone.0236997)
Supplement: Attachment — Submitted filename: 200630ResponseToAcademicEditor.docx [file pone.0236997.s005.docx]

**Author Response to Academic Editor:**

We greatly appreciate your comments on our manuscript. According to your suggestion, we changed the applicable part as follows: from “This might be due to women’s motherly instincts, such as anxiety about childbearing and raising children [7, 8, 13, 14]” to “While no qualitative exploration was done in this particular instance, it may be surmised that women, especially those of childbearing age, may be particularly concerned about the possible effects of radiation on fertility and progeny [7, 8, 13, 14]”. (Page 14, Lines 176 – 179).
